# North Italy: Welcome to the Tropics!

**DOI:** 10.3390/idr13010024

**Published:** 2021-03-05

**Authors:** Federica Veronese, Francesca Graziola, Pamela Farinelli, Elisa Zavattaro, Vanessa Tarantino, Elia Esposto, Paola Savoia

**Affiliations:** 1 Dermatologic Clinic, AOU Maggiore della Carità Hospital, C.so Mazzini 18, 28100 Novara, Italy; francy.gra@tiscali.it (F.G.); pamela.farinelli@gmail.com (P.F.); 2 Dermatologic Clinic, Department of Translational Medicine, University of Eastern Piedmont, Via Solaroli 17, 28100 Novara, Italy; elisa.zavattaro@med.uniupo.it; 3 Dermatologic Clinic, Department of Health Science, University of Eastern Piedmont, Via Solaroli 17, 28100 Novara, Italy; vanessa.tarantino30@gmail.com (V.T.); espostoelia@gmail.com (E.E.); paola.savoia@med.uniupo.it (P.S.)

**Keywords:** larva migrans, autochthonous, creeping eruption

## Abstract

We describe a case of cutaneous *Larva Migrans* in an 8-year-old Caucasian girl. The lesion appeared ten days after a bath in the river in a valley in the north-east of Piedmont. The patient was successfully treated with Albendazole 400 mg daily for 5 days. Autochthonous cases are rare, particularly in northern Italy. Probably the high temperatures and the high degree of humidity favored by the climate changes to which Europe is subjected are favorable to the development of larvae. The diagnosis of cutaneous *Larva Migrans* should, therefore, be considered also in individuals who have not traveled in geographic areas at risk for the climate.

## 1. Case Report

**Figure 1:**
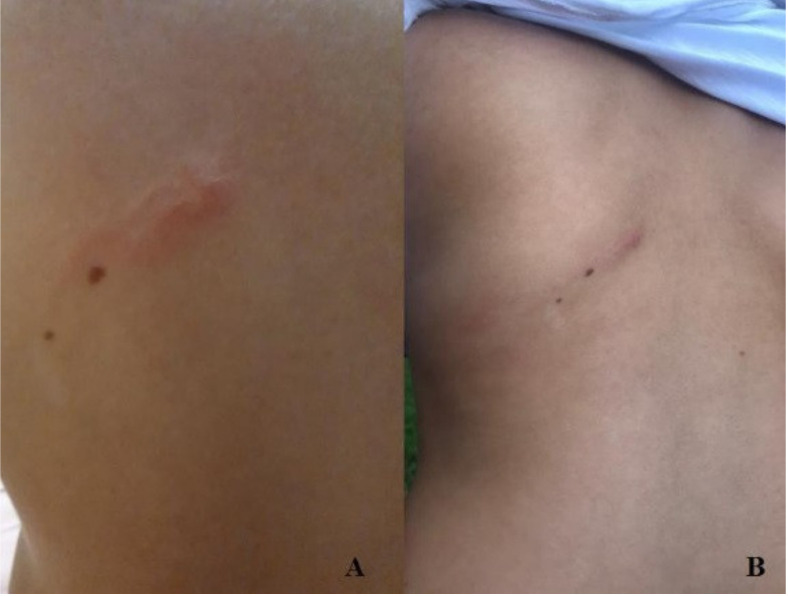
(**A**) Erythematous micropapular rash localized in the subscapularis area. (**B**) Two days later the lesion extended 5–6 cm linearly.

An 8-year-old healthy Caucasian girl presented with an erythematous micropapular rash of 4 cm in the larger diameter, with a linear distribution, localized in the subscapularis area, which arose 24 h earlier (Figure 1A). The patient denied pruritus or pain. In her medical history, the mother reported chickenpox and slept face disease. The girl had not traveled out of Italy recently.

Because of the lack of symptoms, we decided to watch and wait. Two days later the patient presented with a raised and pruritic lesion which extended 5–6 cm linearly (Figure 1B). She had no other similar lesions elsewhere.

Based on the clinical aspect at the onset, our hypothesis was: (i) insect bite with lymphangitis, (ii) lichenoid eruption, (iii) phytophotodermatitis, (iv) herpes zoster or (v) cutaneous *Larva Migrans* (*CLM*).

**Figure 2:**
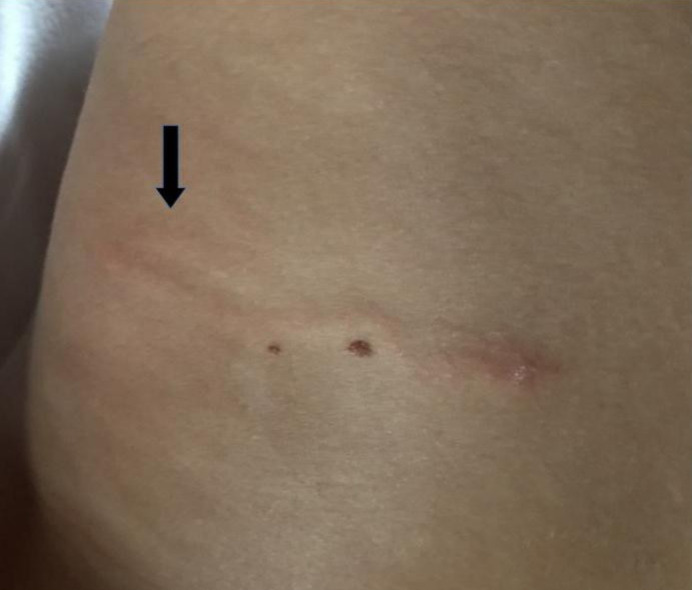
Lesion extended in a curvilinear way (arrow).

Twenty-four hours later, the lesion had extended about another 2 cm in a curvilinear way with persistent pruritus (Figure 2). The patient had not taken any medications in the previous weeks and had not been exposed to the sun recently. However, ten days before the lesion appears, the little girl has taken a bath in the river in a valley in the north-east of Piedmont (district of Verbano-Cusio-Ossola). 

**Figure 3:**
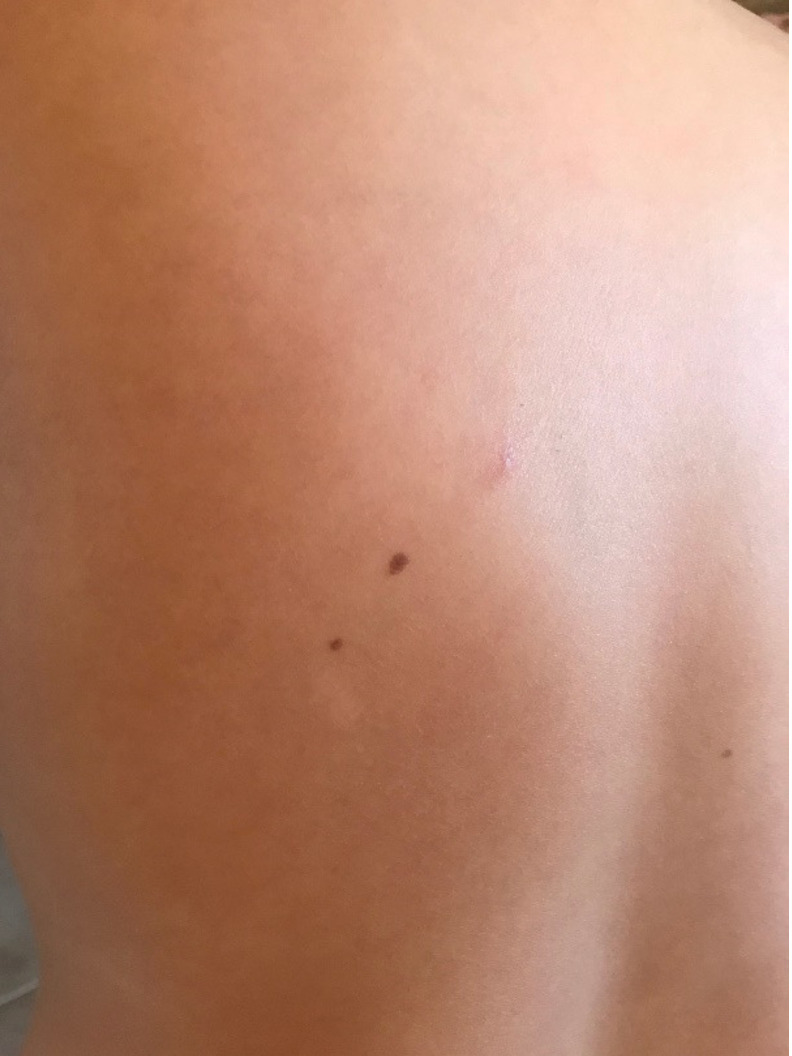
The healing after 5 days of therapy.

Based on these elements we clinically diagnosed CLM and we started treatment with Albendazole 400 mg daily for 5 days (given the young age of the patient we postponed the execution of the skin biopsy). Therapy was well-tolerated and after 5 days the lesion disappeared (Figure 3). The rapid response to the therapy allowed us to definitively confirm the diagnosis.

## 2. Discussion

CLM or “creeping eruption” is a common dermatosis among travelers returning from vacations in warm tropical or sub-tropical countries. This condition is caused by different species of hookworms [1,2]; the most commonly implicated are *Ancylostoma braziliense*, *Ancylostoma caninum*, and *Uncinaria stenocephala*, which are parasites of animals such as cats and dogs [2,3]. Transmission occurs when the hookworm’s eggs pass through the host’s feces to the ground, where, if there are in favorable conditions, they progress to the infective stage (filariform larva) [4,5]. The infectious larvae penetrate the host’s naked skin and, after an incubation period of about 7 days, start migrating [5]. It is considered a self-limiting disease because the larvae cannot penetrate the skin basal membrane and are unable to deeply invade the skin, but progress only in the epidermis [2,5].

Penetration occurs through the fissured skin, but the larvae may penetrate unbroken skin between the stratum germinativum and corneum, perhaps through proteolytic enzymes; penetration through clothing is also possible [6]. The migration advances by mm to several cm each day [6].

Infection sites are principally represented by feet and interdigital spaces of the toes, but in children also buttocks, hands, and knees; however, the habit of covering up less in summer suggests the potential involvement of all anatomic parts [6]. The places at risk are sandy beaches, rivers, lakes, and playgrounds frequented by infected animals, particularly in warm and humid countries [2,3].

Autochthonous cases are rare and isolated, but in the last years are rising in southern Europe [5,7]. In the literature, autochthonous cases have been reported from France, Spain, Portugal, Serbia, Germany, England, and Romania [2,5,8].

The diagnosis is usually clinical and treatment options are represented by oral albendazole, mebendazole, and ivermectin or topical thiabendazole [1]; if untreated, the larvae die in 2–8 weeks, but, rarely, they can persist for up to a year [6].

The treatment is recommended because, even if cutaneous lesions are self-healing, CLM may be associated with systemic involvement. Hypersensitivity reactions with recurrent episodes of urticaria or laryngeal edema and a systemic reaction to soluble larval antigens such as in the Loeffler’s syndrome (pulmonary infiltration with eosinophilia) have been described [6].

## 3. Conclusions

Despite the rarity of autochthonous cases, particularly in northern Italy, our experience suggests that dogs and cats parasitized by nematodes may exist at every latitude in the world; in particular, the high temperatures and the high degree of humidity favored by the climate changes to which Europe is subjected are favorable to the development of larvae [3,5]. The diagnosis of CLM should, therefore, be considered also in individuals who have not traveled in geographic areas at risk for the climate.

## Data Availability

Not applicable.
